# Characterization of myocardial T1-mapping bias caused by intramyocardial fat in inversion recovery and saturation recovery techniques

**DOI:** 10.1186/s12968-015-0136-y

**Published:** 2015-05-10

**Authors:** Peter Kellman, W Patricia Bandettini, Christine Mancini, Sophia Hammer-Hansen, Michael S Hansen, Andrew E Arai

**Affiliations:** National Heart, Lung, and Blood Institute, National Institutes of Health, DHHS, 10 Center Drive MSC-1061, Bethesda, MD 20892 USA

**Keywords:** T1 map, MOLLI, SASHA, Chronic myocardial infarction, Lipomatous metaplasia, Fatty metaplasia, Fat

## Abstract

**Background:**

Quantitative measurement of T1 in the myocardium may be used to detect both focal and diffuse disease processes such as interstitial fibrosis or edema. A partial volume problem exists when a voxel in the myocardium also contains fat. Partial volume with fat occurs at tissue boundaries or within the myocardium in the case of lipomatous metaplasia of replacement fibrosis, which is commonly seen in chronic myocardial infarction. The presence of fat leads to a bias in T1 measurement. The mechanism for this artifact for widely used T1 mapping protocols using balanced steady state free precession readout and the dependence on off-resonance frequency are described in this paper.

**Methods:**

Simulations were performed to illustrate the behavior of mono-exponential fitting to bi-exponential mixtures of myocardium and fat with varying fat fractions. Both inversion recovery and saturation recovery imaging protocols using balanced steady state free precession are considered. In-vivo imaging with T1-mapping, water/fat separated imaging, and late enhancement imaging was performed on subjects with chronic myocardial infarction.

**Results:**

In n = 17 subjects with chronic myocardial infarction, lipomatous metaplasia is evident in 8 patients (47%). Fat fractions as low as 5% caused approximately 6% T1 elevation for the out-of-phase condition, and approximately 5% reduction of T1 for the in-phase condition. T1 bias in excess of 1000 ms was observed in lipomatous metaplasia with fat fraction of 38% in close agreement with simulation of the specific imaging protocols.

**Conclusions:**

Measurement of the myocardial T1 by widely used balanced steady state free precession mapping methods is subject to bias when there is a mixture of water and fat in the myocardium. Intramyocardial fat is frequently present in myocardial scar tissue due lipomatous metaplasia, a process affecting myocardial infarction and some non-ischemic cardiomyopathies. In cases of lipomatous metaplasia, the T1 biases will be additive or subtractive depending on whether the center frequency corresponds to the myocardium and fat being in-phase or out-of-phase, respectively. It is important to understand this mechanism, which may otherwise lead to erroneous interpretation.

## Background

The longitudinal relaxation time constant (T1) of the myocardium is altered in various disease states due to increased water content or other changes to the local molecular environment. Quantitative measurement of T1 in the myocardium may be used to detect both focal and diffuse disease processes such as interstitial fibrosis or edema. Detection of disease at an early stage by measurement of subtle changes requires a high degree of reproducibility [1]. Reproducibility is fundamentally limited by precision and may be further limited by systematic errors [2,3]. A partial volume problem exists when a voxel in the myocardium also contains blood and/or fat. This paper describes how a voxel containing a partial volume of fat and myocardium may affect the estimate of native myocardial T1. This issue stems from the fact that for balanced steady state free precession (b-SSFP) protocols the water and fat signal components can have opposite phase so that the combined signal measured during magnetization recovery represents the difference rather than the sum of the two components.

Artifacts due to partial volume effects between water and fat are well known [4-9] and the out-of-phase cancellation causes a distinctive appearance sometimes referred to as an India ink artifact. A number of studies have proposed exploiting the appearance of this artifact to detect the presence of fat [4,6,7,10]. This approach to detection of fat is limited by the fraction of fat in the voxel and the size of the region which determine the contrast and conspicuity between water and fat. Fat water separated imaging [11-13] provides a positive contrast fat image which improves detectability and objectivity. Fat water separated imaging is limited by the SNR. Fat may also be recognized in T1-maps [14] due to the low T1 of fat provided that the region is sufficiently large. The subject of this present work is the effect of fat on T1-maps in the case of intramyocardial fat where the fat fraction is relatively small. In this situation, the estimated T1 of voxels containing the combination of water and fat may be difficult to simply interpret. We seek to elucidate this complex interaction. The work by Thiesson et al. [15] has independently characterized this mechanism and has explored the quantification of low concentration intramyocardial lipids exploiting the variation of T1 estimates with off-resonance frequency.

Myocardial T1 is most commonly measured with b-SSFP readout using either inversion recovery (IR) or saturation recovery (SR) approaches in which the recovery curve is sampled at various time points, and the T1 is estimated by curve fitting. Current methods such as the MOdified Look-Locker Inversion recovery (MOLLI) sequence [16] or the more recently proposed SAturation recovery with single-SHot Acquisition (SASHA) method [17] use a fit that assumes a single species with mono-exponential recovery. In cases where the voxel contains 2 species with significantly different values of T1, a T1 estimate based on a mono-exponential fit that assumes a single species will not correctly fit the data. Partial volume errors violate the assumptions of most current cardiac T1-mapping methods.

In cases such as tissue boundaries between the myocardium and blood, border voxels containing a partial volume of each will produce a T1 estimate that is an intermediate value between the myocardium and blood in proportion to the amount of myocardium and blood. However, in the case of voxels containing myocardium and fat, the estimate of T1 may actually be higher than either the myocardium or fat and, thereby, may appear as an artifactual elevation of T1. The mechanism for this bias is related to the use of balanced steady state free precession readout and is described in this work. It stems from the fact that for many protocols the water and fat components have opposite phase and the measurement of signal recovery are the difference rather than sum of the individual components.

The importance of this is broader than simply the sub-epicardial myocardium bordering the epicardial fat. Intramyocardial fat is associated with replacement fibrosis due to the process referred to as lipomatous metaplasia, which occurs in both ischemic and non-ischemic scarring. There is a fairly high prevalence of lipomatous metaplasia in chronic myocardial infarction (MI) [18] which can be visualized by water/fat separated imaging [9,11,12]. Intramyocardial fat may also occur in cases of lipomatous hypertrophy and other lipophilic disorders [11]. Since there is an interest in application of native T1 mapping to detecting subtle disease due to fibrosis [19-28], it is important to understand the bias that may be introduced in the presence of intramyocardial fat.

## Methods

### Theory

Water and fat are not in chemical exchange [29,30]. Therefore, any voxel containing a mixture of water and fat will have a bi-exponential recovery curve in proportion to their relative fractions. Curve fitting to a single species with mono-exponential recovery will produce a single T1 value and will result in an error referred to as the partial volume effect.

**Figure 1:**
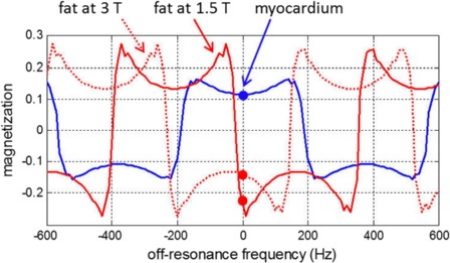
SSFP off-resonance response for myocardium (blue) and fat (red) illustrating the banding effect for typical MOLLI protocol with TR = 2.65 ms and FA = 35 degrees. Note that for this protocol, the MR signal of fat is out of phase with water for both 1.5 and 3T.

Both the MOLLI inversion recovery [16,31] and SASHA saturation recovery [17] method use a b-SSFP readout which has an off-resonance response with alternating polarity of bands [32,33]. The b-SSFP off-resonance response is shown in Figure 1 for a typical MOLLI imaging protocol to illustrate “banding” with periodic changes in signal polarity. In this example, the imaging protocol used a TR = 2.65 ms (corresponding to a readout resolution of 256) and excitation flip angle of 35°, and was calculated on the transient approach to steady state after n = 27 pulses corresponding to the center of k-space [3]. This plot is intended to illustrate that when the myocardium is on-resonance, the fat and myocardium signals have opposite polarities. The chemical shift for fat is approximately 210 Hz at 1.5T and 420 Hz at 3T [34]. The TR used in many b-SSFP based T1-mapping protocols is such that the water and fat components have opposite polarities for normal on-resonance imaging, therefore the apparent signal measured during recovery is combined as the difference rather than sum of the individual components.

Figure 1 also illustrates the degree to which the center frequency (off-resonance) determines the in- and out-of-phase behavior of the myocardial water and myocardial fat signals. At 1.5 T small shifts in the center frequency (> +25 Hz) due to off-resonance shim variation or mis-adjustment may result in the fat being close to nulled or having the same polarity as the myocardium. This will result in a distinctly different mixture of fat and water signals with different apparent T1 recovery. The magnetization recovery curve is sensitive to the specific protocol parameters and scanner adjustment [3]. The actual shape of the off-resonance response will vary for each measurement at different points on the magnetization recovery curve (i.e., inversion time or saturation delay) [35]; however, the frequency bands, which dictate whether the fat and water are added or subtracted, are set by the TR as illustrated in Figure 1 which is calculated near full recovery for the image with longest inversion time.

For shorter TR’s the frequency shift between the on-resonance frequency and the point of sign change 1/(2TR) will become wider. For instance, for a readout resolution of 192 with TR = 2.4 ms, the sign change is at 208 Hz. In this case, the chemical shift of fat at 1.5 T (approx. 210 Hz) is very close to the null point and the sign of the fat will become highly dependent on the shim which will vary spatially across the heart. Some regions of the heart may be in-phase while other regions are out-of-phase. For longer TRs > 3 ms [36], the bands will be narrower and the water and fat will generally be out of phase over a wider frequency range. At 3T with approximately 420 Hz chemical shift, the relative polarity of the fat signal with respect to the myocardium will generally be out-of-phase and will be less dependent on small off-resonance variations. For SASHA with b-SSFP readout with variable flip angle [37] the off-resonance response will be different, but the banding will be in the same locations as determined by TR.

### Simulations

The magnetization was calculated for varying combinations (mixtures) of water and fat for both IR and SR approaches, based on MOLLI [3,16] and SASHA methods [17,37], respectively. The simulation of T1 measurements in this article is based on waveform level Bloch-simulations and curve fitting using the following MOLLI and SASHA protocols. The SSFP readout used a 480 μs low time-bandwidth product Hamming weighted sinc pulse with ≈ 8 mm slice thickness, and TR = 2.65 ms (bandwidth 1085 Hz/pixel), and MOLLI used 5 pulses with linear ramp flip angle to catalyze toward steady state. The matrix (256 × 144) assumed parallel imaging with factor 2 acceleration, separate reference lines, and partial Fourier factor of 7/8 in the phase encoding direction. The actual number of phase encodes was 63 with center at line 27. MOLLI used a tan/tanh adiabatic inversion with 2.56 ms duration, and SASHA used an adiabatic BIR4-90 with 5.12 ms duration. Excitation flip angles were 35° and 70° for MOLLI and SASHA, respectively, with SASHA using a variable flip angle [37] to reduce bias errors in 2-parameter fitting and reduce artifacts due to transient approach to steady state. MOLLI used a minimum TI of 110 ms, and TI increment of 80 ms. The SASHA protocol [17] acquired a fully recovered image plus 9 additional images acquired with saturation times spaced uniformly over the RR interval with minimum “inversion” time of 100 ms. Simulations were performed for a heart rate of 60 bpm. The MOLLI acquisition used a 5(3)3 protocol [3], which is equivalent to the 5s(3s)3s protocol at the HR = 60 bpm. MOLLI used PSIR 3-parameter curve fitting with phase sensitive reconstruction [38] and the conventional Look-Locker correction [16], and SASHA used 2-parameter fitting.

Magnetization transfer was not simulated in the calculation of myocardial signal [39], therefore a myocardial T1 assumed a nominal value of 1000 ms (T2 = 45 ms) in these calculations, whereas the calculations for SASHA used a nominal value of T1 = 1175 ms. Fat was modeled by a single spectral component at −210 Hz (1.5 T) with T1 = 260 and T2 = 60 ms. The proton density fat fraction (FF) was defined as FF = F/(W + F), where W and F are the water and fat signal amplitudes, respectively. T1-estimates were also calculated for in-phase mixtures.

For each magnetization recovery a non-linear curve fit was performed using 3-parameter fitting for MOLLI, S = A – B exp(−TI/T1*), and 2-parameter fitting for SASHA, S = A(1 – exp(−TS/T1)), where TI and TS are the inversion time and saturation time, respectively, and T1* is the apparent longitudinal relaxation time [16]. Recovery curves and fits are shown to illustrate the mismatch between the bi-exponential model of combined water and fat and the fitted mono-exponential model. Magnetization signal recovery curves are plotted as the real part of the transverse magnetization (Mxy) after phase sensitive reconstruction using the phase of the fully recovered image at longest TI [38]. Fitting for MOLLI uses the real part whereas fitting for SASHA is performed on the magnitude data. The estimated values of T1 and MOLLI T1* versus FF are indicated for each plot. Standard deviations reflecting the model mismatch of the curve fits were calculated and plotted versus FF. Standard deviation are calculated based on the fit error [40,41].

### Imaging

Imaging was performed on both 1.5T Siemens MAGNETOM Aera and 3T Siemens MAGNETOM Skyra scanners (Siemens Medical Solutions, Erlangen, Germany). The MOLLI imaging protocol used for native T1 acquired data at 8 inversion times over an 11 heart beat breath-hold at end-expiration with 2 inversions using a 5s(3s)3s scheme [3]. The SASHA protocol used a NS+[(0)1]^12^ sampling [41].

Typical imaging parameters for MOLLI were: non-selective adiabatic inversion pulse [42], steady state free precession single shot read out with 35° excitation flip angle, typical field of view 360 × 270 mm^2^, slice thickness 6 mm, matrix 256 × 144, voxel size 1.4 × 1.9 × 6.0 mm^3^, TR/TE 2.65/1.1 ms, with 200 ms readout imaging duration, minimum inversion time 110 ms, inversion time increment 80 ms, 7/8 partial Fourier plus parallel imaging factor 2.

SASHA used the same FOV and slice thickness, matrix size, TR/TE, and used a variable flip angle (VFA) readout with maximum FA of 70° [37]. SASHA used a composite saturation pulse designed for > 99% saturation efficiency over a wide range of effective transmit flip angles (i.e., < 1% residual magnetization). Non-rigid image registration was used to correct respiratory motion [43]. Both T1 and standard deviation (SD) maps were generated on a pixel-wise basis [40,41].

In addition to T1-mapping, multi-echo gradient recalled echo (GRE) fat-water (FW) separated imaging was performed as well as PSIR late gadolinium enhancement (LGE) [44]. Prior to contrast, FW separated imaging used a dark blood prepared multi-echo sequence with 4 echoes [11], and after contrast, a PSIR FW LGE sequence [12] was acquired as well, if time constraints permitted. Typical parameters for the dark blood FW imaging protocol were: double inversion recovery preparation for blood suppression, GRE with 4 echos and monopolar readout, bandwidth of 977 Hz/pixel, echo spacing 10.4 ms with echo times of 1.6, 3.9, 6.2, and 8.5 ms, matrix size of 256 × 144, 360 × 270 mm^2^ typical FOV, 8 mm slice thickness, 12° excitation flip angle, breath-held, segmented with 20 views per segment for a total acquisition of 9 heart beats per slice including 1 dummy. The PSIR FW LGE sequence was similar to the dark blood breath-held segmented protocol substituting the dark blood preparation with an adiabatic inversion every 2 RR intervals with proton density image acquired on alternate heart beats using a 5° excitation flip angle, and using a 25° excitation flip angle for images acquired following the inversion. In cases for which patients were unable to breath-hold, single shot motion correction averaging was used with 3 echos, parallel imaging factor 3, and 9 repeated measurements. FW image reconstruction was performed using a non-linear least squares formulation using a multi-peak model for fat [34,45]. Field maps displaying the variation of center frequency across the field of view were calculated as a by-product of the fat water separated reconstruction.

In order to demonstrate (n = 2 subjects) the variation of appearance of fat/water mixtures in T1-mapping at different off-resonance frequencies, T1-maps were additionally acquired at an offset center frequency (−150 Hz) corresponding to an in-phase condition.

### Patient studies

This study was approved by the Institutional Review Board of the National Heart, Lung, and Blood Institute, and all subjects gave written informed consent to participate. Patients with chronic MI were recruited prospectively in follow-up of prior acute MI studies. Subjects (n = 17) were at least 4 months following the acute MI. All chronic MI studies were performed on the 1.5 T scanner, and a single normal subject was scanned at 3 T to illustrate the partial volume effect at the epicardial boundary occurring with a larger fat chemical shift. Measurement of the water and fat signal and native T1 were made in the region of MI, and FF were calculated. Presence of fat in the MI was determined from the fat image from the water fat separated imaging.

## Results

### Simulations

**Figure 2:**
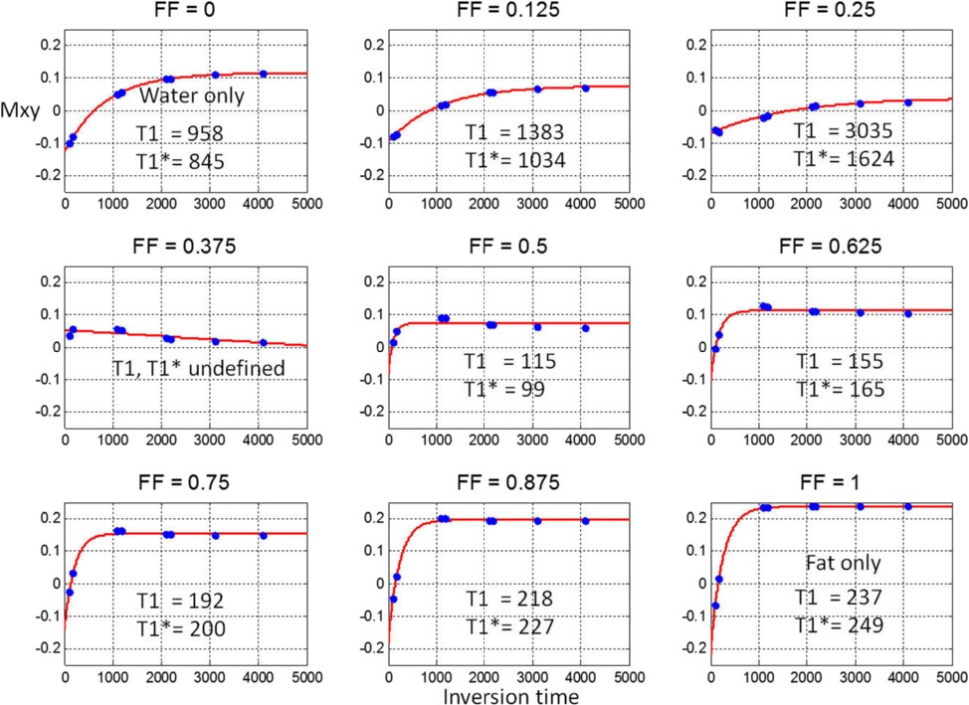
Inversion recovery measurements (circles) for out-of-phase water and fat mixtures at various fat fractions and 3-parameter mono-exponential fits (red) to measurements using MOLLI protocol.

**Figure 3:**
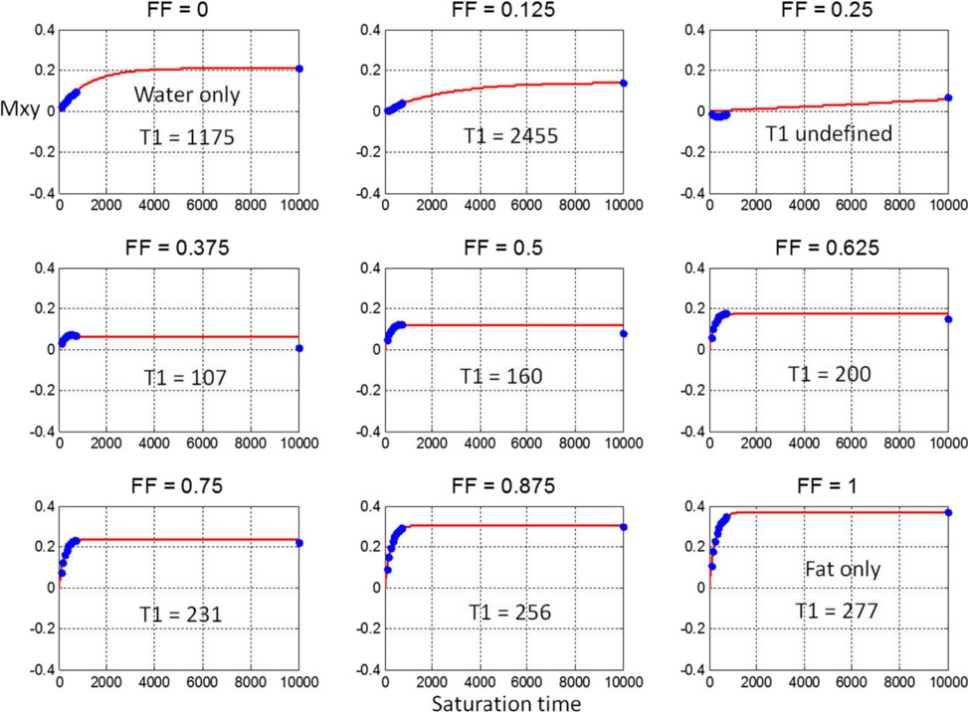
Saturation recovery measurements (circles) for out-of-phase water and fat mixtures at various fat fractions and 2-parameter mono-exponential fits (red) to measurements using SASHA protocol.

The magnetization recovery signal is plotted for water/fat mixtures at varying fat fraction for inversion (Figure 2) and saturation recovery (Figure 3). Note that the T1-estimate of combined water and fat varies substantially with FF. The out of phase fat in the MOLLI recovery curves with high FF > 0.5 appear to be in-phase due to the phase sensitive reconstruction. The circles represent the measurements times of typical MOLLI (Figure 2) and SASHA (Figure 3) protocols, respectively. The red lines are a curve fit for estimating T1 based on a mono-exponential model for a single species. The first and last plots correspond to water only (FF = 0) and fat only (FF = 1), respectively.

**Figure 4:**
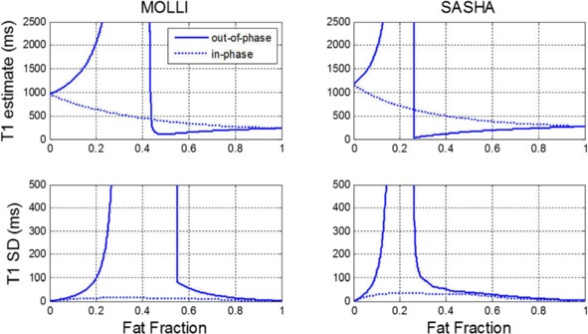
Estimated T1 (top) and SD (bottom) of fat water mixtures vs fat fraction for inversion recovery using MOLLI protocol (left) and saturation recovery using SASHA (right). Solid lines correspond to an out-of-phase mixture of myocardium and fat, and dotted lines are an in-phase mixture.

The estimate of T1 vs fat fraction and the SD due to model mismatch from a mono-exponential are plotted (Figure 4) for MOLLI and SASHA protocols for both out-of-phase (solid) and in-phase (dotted) mixtures of myocardium and fat. For a FF as low as 5%, the T1 is elevated by 60 ms for MOLLI and 80 ms for SASHA, and the T1 elevation is approximately linear with FF in the range of low FF. For FF in the range 30-50% the recovery signal model is an extremely poor fit, and the estimate of T1 is undefined in this range.

### Patient studies

T1-maps using both MOLLI and SASHA methods, with water fat separated imaging in the corresponding slice, were acquired in n = 17 subjects with chronic MI. Subjects were age 58.5 ± 14.8 years (m ± SD) (13 male) ranging from 38 to 88. The mean age of the MI was 3.6 ± 4.4 years (m ± SD) ranging from 4 months to 18 years, where the age of the MI for 1 subject was unknown. MI was determined by clinical history as well as confirmed by LGE. Lipomatous metaplasia was clearly evident in 8 of the 17 subjects (47%) in fat water separated images (FF > 10%). In these cases, the lipomatous metaplasia appeared bright on T1-maps and not dark as one might expect considering the shorter T1 of fat. Lipomatous metaplasia was considered possible in 4 other cases but too subtle for confident assessment. Lipomatous metaplasia was clearly evident in MI as early as 6.5 months. There was no statistical difference in age of MI between the groups with and without lipomatous metaplasia by paired t-test (p = 0.23). For MOLLI, the measured T1 was 1405 ± 242 ms (FF > 10%) and 1080 ± 104 ms (FF < 10%). For SASHA, the measured T1 was 1750 ± 274 ms (FF > 10%) and 1234 ± 79 ms (FF < 10%). The FF was 22.6 ± 6.3% for subjects with lipomatous metaplasia, and 3.9 ± 2.2% for subjects without. The measured T1 for regions remote to the MI was 1032 ± 43 ms for MOLLI and 1209 ± 47 ms for SASHA.

**Figure 5:**
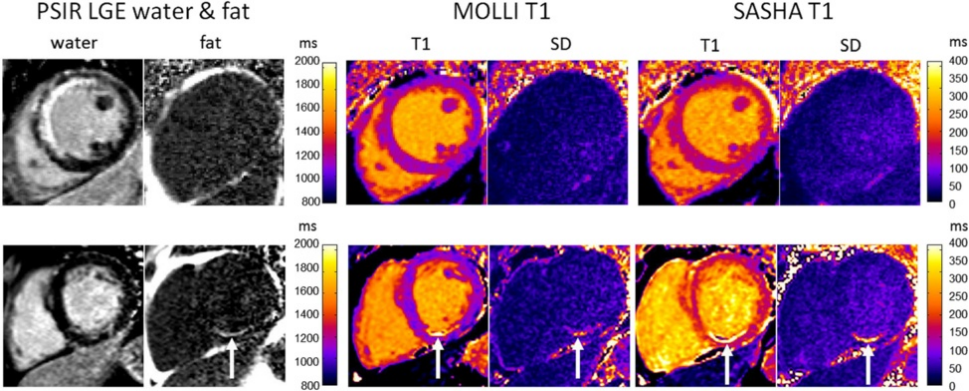
Example of T1-maps for subjects with chronic MI for cases without lipomatous metaplasia (top) and with lipomatous metaplasia (bottom). T1-maps with lipomatous metaplasia (white arrows) have elevated apparent T1 in MI region with out-of-phase mixture of myocardium and fat. The SD in regions of lipomatous metaplasia is elevated due to the model mismatch in fitting single exponential to bi-exponential measurements.

T1-maps for subjects with chronic MI both with and without lipomatous metaplasia are illustrated in Figure 5. T1-maps of lipomatous metaplasia (white arrows) have an elevated apparent T1 in the MI region rather than a shorter T1 expected for fat, explained by the out-of-phase combination of myocardial water and fat as explained in the theory section and simulations. The T1 in the core of the MI region was 2179 vs 1013 ms in remote myocardium for MOLLI, and 2136 vs 1192 ms for SASHA.

The SD in regions of lipomatous metaplasia is elevated due to the model mismatch in fitting single exponential to bi-exponential measurements. The SD for MOLLI was 287 ms in the core of the MI region compared with 38 ms in a remote region nearby, and was 377 vs 45 ms for SASHA.

**Figure 6:**
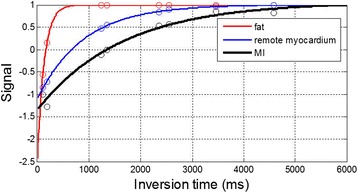
Measured signal for inversion recovery (MOLLI) in chronic MI with out-of-phase mixture (black) for subject with lipomatous metaplasia shown in Figure 5. Signal from regions with pure fat (red) and remote myocardium (blue) are shown for reference. Signals are normalized to fully recovered value.

The fat fraction in the MI core was approximately 33% measured from the native contrast fat water images with measured SNR approx. 8 and 15 for fat and water, respectively, at the location of the MI. The lipomatous metaplasia in this example (Figure 5 bottom) demonstrates the out-of-phase mixture. For this subject, the measured inversion recovery signal (MOLLI) is plotted (Figure 6) for the core of the chronic MI with out-of-phase combination (black), and the region with pure fat (red) (epicardial fat T1 measured as 257 ms) and remote myocardium (blue) are shown for reference.

**Figure 7:**
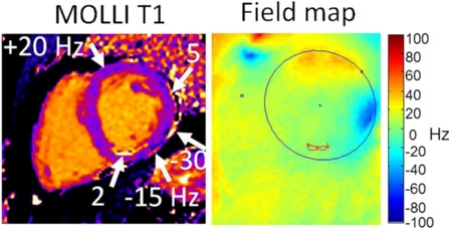
MOLLI T1-map and field map for subject with lipomatous metaplasia shown in Figure 5 illustrating the sensitivity of the partial volume effect to off-resonance. Off-resonance varies across the LV (−60 to +41 Hz). For this protocol (TR = 2.65 ms), out-of-phase mixtures occur for center frequencies below approx. +20 Hz causing fat/myocardium mixture to be bright (elevated T1) in MI region and on myocardium border with sub-epicardial fat on lateral wall, but are in-phase (or nulled) for center frequency CF > 20 Hz as seen in the anterior region.

The field map (Figure 7) shows the variation in off-resonance across the heart. The average center frequency is 4.4 ± 17.7 Hz (m ± SD) across the LV, ranging from −60 to 41 Hz. For this protocol (TR = 2.65 ms), the fat will be approximately nulled for center frequency (CF) = 20 Hz, will be an out-of-phase combination for CF < +20 Hz, and will be an in-phase combination for CF > +20 Hz. The T1 appears bright (artifactually elevated T1) for the out-of-phase mixture in MI region (CF = 2 Hz), and on the myocardium border with epicardial fat on lateral wall (−30 < CF < 5 Hz), but is in-phase (or nulled) in the anterior region (CF ≥ 20 Hz).

**Figure 8:**
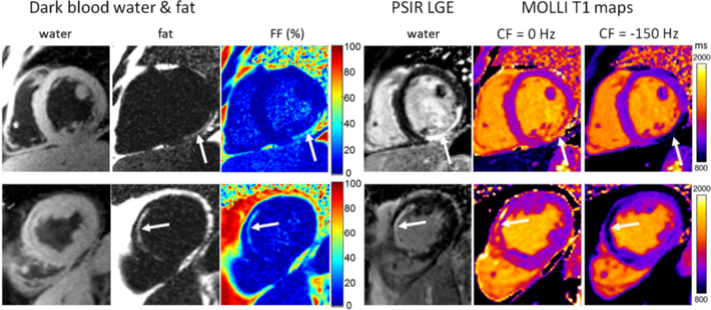
Example of off-resonance dependence of MOLLI T1-map for 2 subjects. Subject 1 (top) with chronic MI at 7.5 months following acute MI and subject 2 is chronic MI at 42 months. Lipomatous metaplasia has a fat fraction in the range of 20-35%. An out-of-phase mixture occurs on resonance causing elevated apparent T1 and an in-phase mixture occurs for CF offset intentionally adjusted to −150 Hz causing an apparent dark T1.

The appearance of lipomatous metaplasia in the T1-map for chronic MI depends on whether the off-resonance creates an in-phase or an out-of-phase mixture. MOLLI T1-maps are shown (Figure 8) for 2 subjects with lipomatous metaplasia for on-resonance (out-of-phase), which leads to a bright apparent T1, and intentionally adjusted −150 Hz off-resonance (in-phase) leading to a dark apparent T1. The measured T1 for the out-of-phase on-resonance condition was 1510 ms and 1423 ms for the 2 subjects, displayed top and bottom, respectively. The measured T1 for the in-phase off resonance condition was 640 ms and 718 ms, respectively. The fat fraction in the core region is in the 20-35% range for both subjects, as seen in the FF maps (Figure 8). The subject 1 study (top) was 7.5 months following the acute MI event and subject 2 (bottom) was at 42 months post MI.

**Figure 9:**
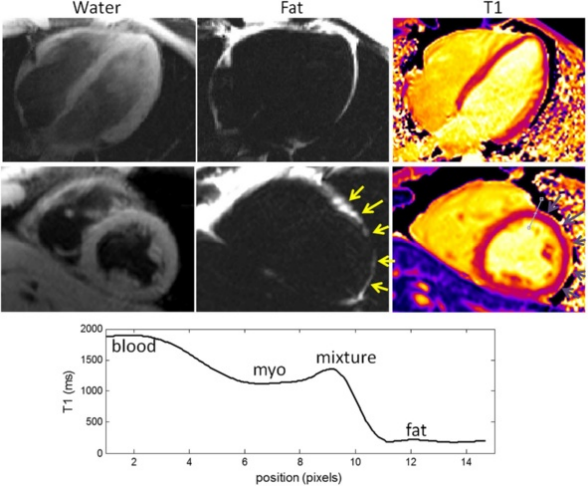
Example of T1 map at 3 T for normal subject. The chemical shift for fat is 420 Hz > 1/2TR resulting in an out-of-phase partial volume mixture across the entire heart despite off-resonance variation due to shim, as evidenced by bright T1 at myocardial border with epicardial fat. The profile of T1 across an anterior profile shows the elevated T1 at the boundary with fat.

At 3T, the chemical shift of fat (420 Hz) is larger than at 1.5T and the fat is generally out-of-phase with the myocardium over the entire heart, even in the presence of off-resonance variation due to shim. An elevated T1 at the boundary between myocardium and epicardial fat will be apparent due to the partial volume mixture at the boundary (Figure 9).

## Discussion

### Partial volume effect

T1 measurement by inversion and saturation recovery methods such as MOLLI and SASHA assume a single species (e.g., myocardium, blood, or fat) and perform a mono-exponential curve fit to derive a single T1 value. Voxels containing a partial volume mixture of myocardium and fat occur at tissue boundaries and in the case of lipomatous metaplasia, which is commonly seen in replacement fibrosis such as chronic MI. The presence of fat leads to a bias in T1 measurement. The mechanism for this artifact and the dependence on off-resonance frequency are described in this paper. The bias is dependent on the fat fraction, the sequence TR, off-resonance, and field strength.

### Lipomatous metaplasia

The prevalence of lipomatous metaplasia in replacement fibrosis is quite high and increases with the time since the clinical injury [18]. The process of lipomatous metaplasia begins immediately after infarction but is often not detectable immediately due to the limitations of sensitivity for detecting intra-myocardial fat. Current methods use spin echo imaging with and without fat saturation. This method relies on negative contrast that is difficult to detect in the presence of other image signal variation [11]. In this study, lipomatous metaplasia was detectable using both fat-water separated imaging as well as by observing the artifactually elevated T1 and clues to poor mono-exponential fits as suggested by the corresponding SD maps. Using fat water separated imaging fat fractions on the order of 5-10% are readily detected. In the example of Figure 8 with 7.5 month old MI, the region of lipomatous metaplasia had a FF of 30-35%. At low values of FF, the drop in myocardial signal in the water only image provides a fairly low contrast that is more subjective to detect in the presence of other signal inhomogeneities. The water image from the water fat separated image reconstruction is equivalent to a well fat saturated image used in conventional imaging protocols for fat detection.

Using T1-mapping where the fat and myocardium are out-of-phase, the T1-elevation for FF of 5% (120 ms and 250 ms for MOLLI and SASHA, respectively) should be detectable. The SD of T1 at an SNR = 20 is 45 ms for MOLLI [40] and 80 ms for SASHA [41] as measured on a pixel-wise basis. Therefore, in the case of 5% FF and SNR = 20, the elevated T1 will be almost 3 SD on a pixel wise basis for both MOLLI and SASHA protocols which is readily detectable. The improved sensitivity and positive contrast of these methods may explain the higher prevalence and lower age MIs exhibiting lipomatous metaplasia compared to that reported in the literature. Even lower lipid concentrations of intracellular fat droplets in the 0.5 to several percent are sufficient to cause T1 measurement biases and may explain some observed variation in T1 measurements.

Previous studies of native T1 in chronic MI have shown elevated T1 in the MI region compared to remote myocardium [23,46-48]. Messroghli et al. [47] reported approx. T1 elevation of 80 ms in chronic MI over remote at 1.5 T in 24 subjects at 6 months post MI. Kali et al. [23], reported 89 ms (1.5 T) and 239 ms (3 T) elevation of chronic MI over remote in canine models (n = 29) at 4 months post MI. Bauner et al., [46] reported 159 ms elevation (1.5 T) of chronic MI over remote in 26 subjects at 6 months post MI. Okur et al., [48] reported T1 elevation of 215 ms (3 T) of chronic MI over remote in 29 subjects with MI at least 6 months following infarction. In the present study reported here, the T1 (at 1.5T) of chronic MI was elevated 49ms above remote in cases with FF <10% and 374 ms for cases with FF > 10%. Previous studies have not reported on fatty infiltration or described specifically whether infiltrative fat might have led to T1 measurement biases. The 3 prior studies performed at ≤6 month post MI [23,46,47] showed less T1 elevation which is consistent with less lipomatous metaplasia. It is not clear how these previous findings may have been affected by lipomatous metaplasia but the present work suggests that some of the apparent elevated T1 may be explained by partial volume errors due to lipomatous metaplasia.

### Other T1-mapping protocols

The present study describes the mechanism of T1 measurement error that results from mono-exponential fitting of inversion or saturation recovery to a mixture of water and fat. The simulations and results are provided for MOLLI and SASHA which are widely used T1-mapping protocols based on b-SSFP with specific strategies for sampling the magnetization recovery signal. The mechanism generally applies to other T1-mapping protocols such as ShMOLLI [49]. For inversion recovery using GRE readout, the polarity of the fat relative to the myocardium will be determined by the echo time (TE) and will be insensitive to off-resonance. Reported GRE based protocols for IR-cine [50] based T1-measurement use TE close to the fat null (1.3 ms at 1.5 T), which partially nulls the fat but also results in an out-of-phase (difference) mixture. Depending on the TE, the degree of fat suppression will determine how large a partial volume error in T1 will result.

## Conclusions

Measurement of the myocardial T1 by current T1-mapping methods that use b-SSFP protocols is subject to bias when there is a mixture of myocardium and fat. Partial volume of myocardium and fat occurs at tissue boundaries and in cases of intramyocardial fat. Intramyocardial fat is frequently present in scar tissue such as chronic MI due to the process of lipomatous metaplasia, and is also present in non-ischemic cardiomyopathies. In cases of lipomatous metaplasia, the T1 biases in b-SSFP imaging protocols will be additive or subtractive depending on whether the center frequency corresponds to the myocardium and fat being in-phase or out-of-phase, respectively. It is important to understand this mechanism which may otherwise lead to erroneous interpretation.

## Abbreviations

b-SSFP: Balanced steady state free precession
CF: Center frequency
CMR: Cardiovascular magnetic resonance
FF: Fat fraction
GRE: Gradient recalled echo
IR: Inversion recovery
LGE: Late gadolinium enhancement
LV: left ventricle or left ventricular
MOLLI: MOdified Look-Locker Inversion recovery
SASHA: SAturation recovery with single-SHot Acquisition
ROI: Region-of-interest
SD: Standard deviation
SR: Saturation recovery
